# Bioinformatics analysis of Rab GDP dissociation inhibitor beta and its expression in non-small cell lung cancer

**DOI:** 10.1186/s13000-014-0201-0

**Published:** 2014-11-04

**Authors:** Zongjuan Ming, Chunli Guo, Meihua Jiang, Wei Li, Yuping Zhang, Na Fan, Yujie Zhong, Xia Meng, Shuanying Yang

**Affiliations:** Department of Respiratory Medicine, the Second Affiliated Hospital of Xi’an Jiaotong University, Xi’an, 710004 China; Department of Respiratory Medicine, People’s Hospital of Tongchuan City, Tongchuan, 727000 China

**Keywords:** Rab GDIβ, NSCLC, Bioinformatics, Expression

## Abstract

**Background:**

Lung cancer has been considered as one of the most important causes of cancer-related mortality worldwide. To predict lung cancer, researchers identified several molecular markers. However, many underlying markers of lung cancer remain unclear. One of these markers is Rab GDP dissociation inhibitor beta (GDIβ), which is related to tumorigenicity, development and invasion. This study was designed to analyze the biological characteristics of Rab GDIβ and to detect the mRNA and protein expressions of Rab GDIβ in lung cancer cells; this study also aimed to investigate the functions of this protein in lung cancer.

**Method:**

Using online software from the websites of NCBI, ProtParam and so on, we analyzed the biological characteristics of Rab GDIβ. RT-PCR was performed to detect gene expressions in A549 and 16HBE cell lines and immunohistochemistry (IHC) staining was conducted to detect Rab GDIβ protein expression in 57 cases of human lung cancer tissues and 19 cases of normal lung tissues. The association of protein expression with patient clinical and pathological characteristics was assessed in each dataset.

**Results:**

Bioinformatic analysis on Rab GDIβ: The mRNA of human Rab GDIβ contains two transcript variants; the common structural elements of the two proteins are mainly α-helix, random coil, β-turn and extended strand. Three and four transmembrane domains could be found in the entire polypeptide chain of protein variants 1 and 2, respectively; both transcript variants are hydrophilic and soluble proteins. The RT-PCR result: The mRNA expression of Rab GDIβ was down-regulation in A549 cells compared with that in 16HBE cells. The IHC result: The protein expression of Rab GDIβ in lung cancer cells was significantly lower than that in normal lung tissues (P <0.05) but was not correlated with patients’ age, gender, tumor size, pathological type, differentiation, lymph node metastasis, distant metastasis and TNM stage.

**Conclusion:**

The expression of Rab GDIβ was low in non-small cell lung cancer (NSCLC). Hence, Rab GDIβ may be a tumor suppressor and could function as an indicator of tumorigenesis in NSCLC; nevertheless, this result should be further studied.

**Virtual Slides:**

The virtual slide(s) for this article can be found here: http://www.diagnosticpathology.diagnomx.eu/vs/13000_2014_201

## Background

Lung cancer has been considered as one of the most common malignancies and yields the lowest survival rate among other cancers [1]. Non-small cell lung cancer (NSCLC) accounts for more than 80% of all lung cancers. Mortality related to this malignant disease has increased by 465% during the last 30 years in People’s Republic of China [2]. Even those NSCLC patients have received standard treatments, including surgical resection, traditional chemotherapy, radiation therapy and molecular targeted therapy, the five-year survival rate of NSCLC is still lower than 15% [3-5]. It has long been acknowledged that the aggressive nature of lung cancer is closely related to the activation of oncogenes and the inactivation of tumor suppressor genes [6-8]. However, numerous molecular alterations are involved in lung cancer development [9]; cancer initiation, progression and metastasis also remain poorly understood. Thus far, we still lack markers that can be used in early detection and targeted therapy. Therefore, novel cancer-specific molecular targets and signalling pathways should be developed to establish new therapeutic strategies against this devastating malignancy and to improve patient survival.

In our previous study on mitochondria proteomics, different proteins containing Rab GDP dissociation inhibitor beta (GDIβ) were screened and identified [10]. Rab GDIβ is a member of the GDP dissociation inhibitor family that controls the recycling of Rab GTPases involved in membrane trafficking [11,12]. Rab GTPases, one of the Ras superfamily members of monomeric GTPases, are small G proteins. In recent years, a new function of Rab proteins has been observed in the control of tumor progression. Evidence [13-19] has further shown that Rab proteins are necessary to facilitate cancer cell adhesion and migration. As a Rab protein control factor, Rab GDIβ is also involved in the development of multiple tumors. Rab GDIβ controls the access of GTPases to regulatory guanine nucleotide exchange factors and GTPase-activating proteins [20]; Rab GDIβ may also function in tumor cell apoptosis [21]. Wang et al. [22] discovered that Rab GDIβ was significantly up-regulated in the highly metastatic gallbladder carcinoma cells GBC-SD18H compared with the poorly metastatic GBC-SD18L cell line. Proteomic analysis results have also shown that Rab GDIβ expressions in gastric cancer [23] and ovarian cancer [24] were aberrant compared with those in normal tissues. Therefore, Rab GDIβ possibly participates in cancer initiation and progression.

However, whether Rab GDIβ is involved in the development of NSCLC is yet to be reported. In the present study, the biological characteristics of Rab GDIβ were analyzed. RT-PCR was conducted to detect the mRNA expression levels of Rab GDIβ in lung adenocarcinoma cell line A549 and normal human bronchial epithelial cell line 16HBE. Immunohistochemistry (IHC) was performed to quantify the protein expression of Rab GDIβ in lung cancer tissues. Then the relationship between this expression and clinicopathological factors was examined. These studies may provide important references related to the potential function of Rab GDIβ in human NSCLC progression.

## Methods

### Bioinformatics analysis

Biological characteristics, including physicochemical properties, homology, secondary structure, transmembrane domain, functional domain, subcellular localisation, three-dimensional (3D) structure, phosphorylation sites and functions of Rab GDIβ, were analyzed using online software from the websites of NCBI, ProtParam, SOPMA, TMpred, SMART, ProtScale, SOSUI, PSORT II, Swiss-MODEL Repository and so on. The gene and protein interaction networks of Rab GDIβ were established on the basis of the platform of GeneMANIA and STRING9.0.

### Cell culture

Human lung adenocarcinoma cells A549 were obtained from the central laboratory of Xi’an Jiaotong University and grown in a complete medium containing RPMI 1640 supplemented with 10% foetal bovine serum and pen/strep (100 U/ml penicillin and 100 U/ml streptomycin). The cells were grown at 37°C in an incubator with a humidified atmosphere of 5% CO_2_ until confluency was reached. Human normal bronchial epithelial cells 16HBE were kindly provided by the tumor Cell Library of the Chinese Academy of Medical Sciences.

### Patients and tissue procurement

57 patients with primary lung cancer who underwent surgical resection at the Department of Thoracic Surgery of the Second Affiliated Hospital of Xi’an Jiaotong University, between June 2006 and June 2011, without pre-operative chemotherapy and/or radiation therapy, were enrolled in this study. 19 cases of normal lung tissues were benign lung lesions or at least 5 cm distant from the cancer site. The collections of lung cancer were mainly NSCLC (32 adenocarcinomas, 19 squamous carcinomas, 2 adenosquamous carcinoma, 1 carcinoid, 1 small cell lung cancer (SCLC), 1 pulmonary metastatic tumor from the oesophagus and 1 pulmonary metastatic tumor from the cervix). Patients’ characteristics, such as gender, age, pathological pattern, lymph node invasion and Union for International Cancer Control (UICC) stage, are summarised in Table 1. The tissue procurement protocol used in this study was approved by the Human Research Committee of Xi’an Jiaotong University, and a written informed consent was obtained from each patient. All of the fresh tumor specimens and normal lung tissues were collected in the operating room, snap frozen in liquid nitrogen and stored at −80°C until analysis.

**Table 1:** **Correlation between clinicopathological characteristics and Rab GDIβ expression**

**Variables**	**Patients [n(%)]**	**Positive**	**Negative**	**Positive rate (%)**	**P**
**Age**					
≥65	31	8	23	25.8	0.740
<65	20	6	14	30.0	
**Gender**					
Male	27	7	20	25.9	0.791
Female	24	7	17	29.2	
**Tumor size (cm)**					
≥3.5	26	7	19	26.9	0.920
<3.5	25	7	18	28.0	
**Histology**					
Adenocarcinoma	32	9	23	28.1	0.889
Squamous carcinomas carcinomas	19	5	14	26.3	
**Differentiation**					
G1-G2	30	8	22	26.7	0.888
G3	21	6	15	28.6	
**Lymph node metastasis**					
Node-positive	29	7	22	24.1	0.543
Node-negative	22	7	15	31.8	
**Distant metastasis**					
Yes	4	2	2	50.0	0.300^※^
No	47	12	35	25.5	
**TNM stage**					
I ~ II	24	7	17	29.2	0.796
III ~ IV	27	7	20	25.9	

^※^Fisher's exact test was used.

### Primary reagent

RPMI 1640 and foetal bovine serum were purchased from Hyclone (USA). RNAfast200 and RevertAid™ first-strand cDNA synthesis kit were separately obtained from Feijie Biotechnology Company (Shanghai, China) and Fermentas (USA). Rabbit polyclonal antibody against Rab GDIβ was purchased from ProteinTech (USA).

### RT-PCR

Total RNA was extracted from cultured cells with RNAfast200 according to the manufacturer’s instructions. A total of 1 μg of RNA was used for reverse transcription; cDNA was generated and used as a template for RT-PCR analysis. RT-PCR was then performed using the RevertAid™ first-strand cDNA synthesis kit. PCR conditions for all of the reactions were as follows: Rab GDIβ, 94°C for 3 min, 94°C for 30 s, 50°C for 30 s and 72°C for 1 min (30 cycles) and 72°C for 5 min; and β-actin, 94°C for 3 min, 94°C for 30 s, 58°C for 30 s and 72°C for 1 min (30 cycles) and 72°C for 5 min. Gel images were collected by applying conventional electrophoresis on the PCR product. The expression levels of Rab GDIβ in A549 and 16HBE cell lines were evaluated using ImageJ 1.44 software and under the reference of β-actin. Each experiment above-mentioned was performed in triplicate.

### Immunohistochemistry

IHC staining was conducted according to standard streptavidin-perosidase (SP) methods. In brief, the tissue specimens were fixed in neutral buffered formalin and then embedded in paraffin wax. Tissue sections (thickness = 4 μm) were dewaxed, rehydrated, subjected to heat-induced antigen retrieval and blocked with normal goat serum for 15 min. The slides were incubated with rabbit polyclonal antibody against Rab GDIβ at 4°C overnight, rinsed with phosphate buffered saline (PBS) and incubated with horseradish peroxidase-labelled secondary antibody. Rab GDIβ localisation was revealed using 3,3ʹ-diaminobenzidine (DAB) as a chromogen. Negative control experiment was performed by replacing the primary antibody with PBS. The IHC staining levels of Rab GDIβ were assessed using a semi-quantitative staining index method [25]. The percentage of positive cells was assessed quantitatively and scored as follows: 0, <5% of the total counted cells were stained; 1, 5% to 24% of the total counted cells were stained; 2, 25% to 50% of the total counted cells were stained; and 3, >50% of the total counted cells were stained. Intensity was graded as follows: 0, no signal; 1, weak; 2, moderate; and 3, strong staining. A staining index ranging from 0 to 6 was generated by multiplying the percentage of positive cells and staining intensity of each sample. The total staining score was also graded as negative (–, score 0), weak (+, score 1 to 2), moderate (++, score 3 to 4), or strong (+++, score 5 to 6). “–” was defined as negative expression, and “+, ++, +++” were defined as positive. All of the slides were examined and scored independently by two pathologists who were blinded from the patient data.

### Statistical analysis

The associations between IHC staining and clinicopathological factors were examined by *χ*^2^ test and Fisher’s exact test. Statistical analysis was performed using the Statistical Package for the Social Sciences (SPSS) 19.0 software, and P <0.05 was considered statistically significant.

## Results

### Biological characteristics of Rab GDIβ

**Figure 1:**
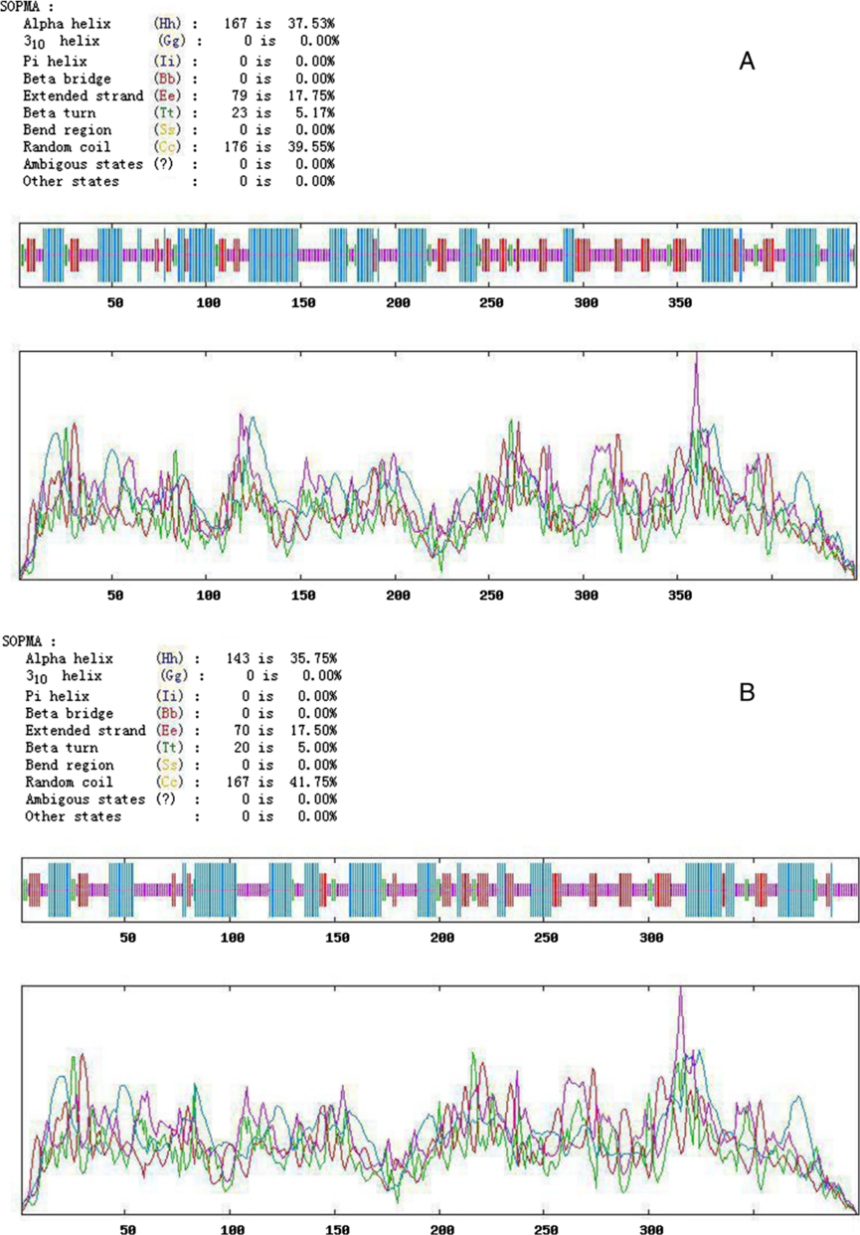
**Analysis of the secondary structure of Rab GDIβ (A, protein variant 1; B, protein variant 2).**

**Figure 2:**
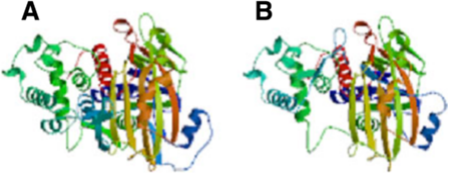
**Prediction of the 3D structure of Rab GDIβ (A, protein variant 1; B, protein variant 2).**

**Figure 3:**
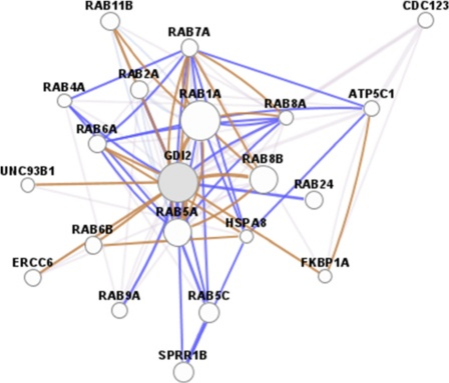
**Network diagram of Rab GDIβ gene interaction.**

**Figure 4:**
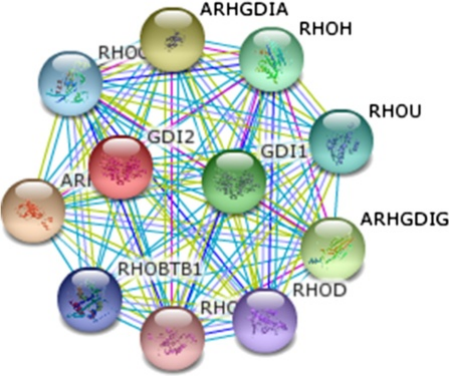
**Network diagram of Rab GDIβ protein interaction.**

Rab GDIβ gene is located at chromosome 10p15 and measures a total length of 48327 bp. The mRNA of human Rab GDIβ exhibits two transcript variants. Transcript variant 1 is 2441 bp long; the initiation codon of the gene is ATG and the termination codon is TAA. The open reading frame of this variant ranges from 292 bp to 1629 bp. Furthermore, transcript variant 1 contains 11 exons and 10 introns. Transcript variant 2 is 2306 bp long. The initiation codon of the gene is ATG and the termination codon is TAA. The open reading frame of this variant ranges from 292 bp to 1494 bp. Transcript variant 2 also contains 10 exons and 9 introns. A high degree of genetic homology is observed in human, gibbon, wren and macaque. For instance, transcript variant 1 is composed of 445 amino acids with a relative molecular mass of approximately 50663.2D; the theoretical isoelectric point and the half-life of this variant are 6.10 and 30 h, respectively. Transcript variant 2 is composed of 400 amino acids with a relative molecular mass of approximately 45619.4D; the theoretical isoelectric point and the half-life of this variant are 5.91 and 30 h. The common structural elements of these two proteins are mainly α-helix, random coil, β-turn and extended strand (Figure 1). Furthermore, three and four transmembrane domains can be found in the entire polypeptide chain of protein variants 1 and 2, respectively. Both variants are hydrophilic and soluble proteins. One and two GDI activity regions are also found in protein variants 1 and 2, respectively. The subcellular localisation of protein variant 1 was analysed and revealed that this protein may be present in the following organelles: 39.1% in the cytoplasm; 17.4% in the mitochondria; 17.4% in the nucleus; 13.0% in the endoplasmic reticulum; 4.3% in the secretory vesicles; 4.3% in the Golgi apparatus; and 4.3% in the peroxisome. Likewise, the subcellular localisation of protein variant 2 was analysed and revealed that this protein may be present in the following organelles: 39.1% in the cytoplasm; 21.7% in the nucleus; 17.4% in the mitochondria; 8.7% in the endoplasmic reticulum; 4.3% in the secretory vesicles; 4.3% in the Golgi apparatus; and 4.3% in the peroxisome. The 3D structure of Rab GDIβ protein was predicted using Swiss-MODEL Repository software (Figure 2). The network diagram of Rab GDIβ gene interaction was obtained using GeneMANIA software (Figure 3). The network diagram of Rab GDIβ protein interaction was constructed using STRING9.0 software and by imposing restrictions on the search conditions within 20 genes (Figure 4).

### mRNA levels of Rab GDIβ in A549 and 16HBE cell lines

**Figure 5:**
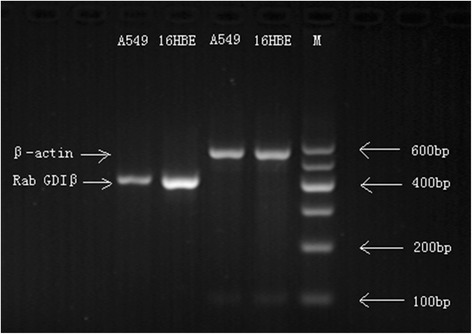
**Electrophoresis of the Rab GDIβ gene PCR product in A549 and 16HBE cell lines (M: marker).**

The electrophoresis results of the mRNA level of Rab GDIβ in A549 and 16HBE cell lines are shown in Figure 5. In particular, the mRNA level of Rab GDIβ in A549 was down-regulated. Using ImageJ 1.44 software and β-actin as a reference, we found that the relative expression level of Rab GDIβ in A549 was 0.25 ± 0.07.

### Protein expression of Rab GDIβ in tissue specimens

**Figure 6:**
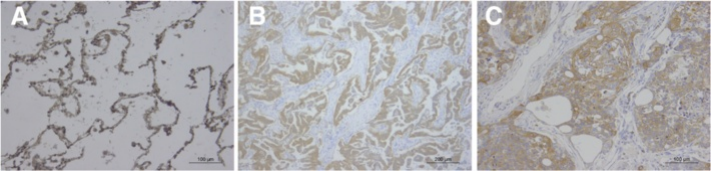
**Expressions of Rab GDIβ in normal lung tissue (A, IHC, 200×), adenocarcinoma (B, IHC, 100×) and squamous carcinomas (C, IHC, 200×).**

Human lung cancer and normal lung tissue specimens were immunohistochemically stained. Our results showed that Rab GDIβ was expressed in the cell membrane and the cytoplasm, as indicated by brown granules (Figure 6). The IHC results also showed that 12 of 19 (63.2%) normal lung samples were positively stained. Among the 57 lung cancer cases, only 18 showed positive expression, and the positive rate was only 31.6%. The protein expression level of Rab GDIβ in adenocarcinoma was uniformly and significantly decreased (P = 0.014) compared with normal samples. Similarly, *χ*^2^ test showed that the P-value between squamous carcinomas and normal tissues is 0.022, meaning that the expressions in squamous carcinomas and normal tissues were also significantly different. Thus, Rab GDIβ expression was either absent or decreased in NSCLC.

### Associations between IHC staining and clinicopathological factors

The Rab GDIβ protein expression in NSCLC tissues was not correlated with the patients’ clinicopathological characteristics, such as age, gender, tumor size, pathological type, differentiation, lymph node metastasis, distant metastasis and tumor node metastasis (TNM) stage (Table 1**)**. Adenosquamous carcinoma, carcinoid, SCLC and metastatic carcinoma were removed during the analysis because the included cases were very few.

## Discussion

Bioinformatics is a new discipline that combines computer techniques and applied mathematics; bioinformatics is also a major core area of life science and natural science. In the current study, bioinformatics was applied to analyze the biological characteristics of Rab GDIβ. Our result revealed high homology in humans and other species, suggesting that Rab GDIβ is conservative and implicated in in vivo processes. Rab GDIβ is primarily located in the cytoplasm and the organelles of membrane structures; this result indeed showed that Rab GDIβ was involved in cellular vesicle transport. Gene and proteins interacting with Rab GDIβ are mainly family members of small G proteins. We could take these data to analyze the biological process and signal transduction pathways which Rab GDIβ may participate in. Thus, bioinformatics provided relevant information to elucidate the structure and function of Rab GDIβ.

Rab GDIβ translocates prenylated Rab proteins from the cytosol to the membrane to form budding transport vesicles. Rab GDIβ also assists the subsequent retrieval of Rab proteins [26,27]; some of these proteins are tumorigenic or tumor suppressive [28]. Although the functions of Rab proteins in cancer progression have been studied intensively, information on Rab GDIβ action in this regard remains limited. Thus far, few expression studies have suggested that Rab GDIβ can activate or inhibit tumor progression. Sun et al. [29] conducted IHC and western blot analyzes, confirming that the increased level of GDIβ is associated with pancreatic carcinoma. In another study, proteomic analysis result has shown that GDIβ is identified as an up-regulated protein with the effect of retinoic acids on the human breast cancer cell line MCF-7 [30]. By contrast, the expression levels of GDIβ in SKpac cells and chemo-resistant human ovarian cancer tissues are down-regulated [24].

In the current study, the mRNA concentrations of Rab GDIβ of lung adenocarcinoma cells and normal human bronchial epithelial cells were quantified by RT-PCR. This study is the first to report that the expression level of Rab GDIβ in lung adenocarcinoma cells was significantly lower than that in normal cells. Considering that mRNA expression may not accurately reflect protein level, we detected protein levels by IHC to verify this conclusion. The results showed that the protein level of Rab GDIβ varied with the corresponding mRNA level of Rab GDIβ in NSCLC and normal tissues; however, the protein level was not associated with patients’ age, gender, tumor size, pathological type, differentiation, lymph node metastasis, distant metastasis and TNM stage. These findings suggested that the expression of Rab GDIβ in NSCLC was low, and this protein may be a candidate biomarker that could be used to diagnose NSCLC in early stages. This protein could also be used to provide new insights into the pathological mechanisms of tumor formation and development.

In a previous study, Rab GDIβ is considered as a gene that promotes differentiation and apoptosis but inhibits proliferation in various tumors. The current study suggested that Rab GDIβ was associated with human NSCLC. Rab GDIβ could be a potentially valuable prognostic indicator in patients with NSCLC. This information may also be used as reference by clinicians when they provide individualised therapy with optimal benefits for patients with NSCLC.

## Conclusion

In summary, our data showed for the first time that the expression of Rab GDIβ decreased in human NSCLC. Rab GDIβ level was not correlated with patients’ age, gender, tumor size, pathological type, differentiation, lymph node metastasis, distant metastasis and TNM stage. Rab GDIβ may be used as a novel marker in early-onset human NSCLC. However, the current study is only a preliminary report, and the number of samples in this research is limited; thus, further experiments should be conducted to confirm our conclusion. We recommend that a larger sample size should be used in future studies, and a cell excessive expression vector should be established to investigate the specific functions of Rab GDIβ in NSCLC.

## Abbreviation

Rab GDIβ: Rab GDP dissociation inhibitor beta
NSCLC: non-small cell lung cancer
IHC: immunohistochemistry
3D: Three-dimensional
SCLC: Small cell lung cancer
UICC: Union for International Cancer Control
SP: Streptavidin-perosidase
PBS: Phosphate buffered saline
TNM: Tumor node metastasis
